# Detection of recurrent Cushing’s disease: proposal for standardized patient monitoring following transsphenoidal surgery

**DOI:** 10.1007/s11060-014-1508-0

**Published:** 2014-07-01

**Authors:** Alejandro Ayala, Alex J. Manzano

**Affiliations:** Division of Endocrinology, Miller School of Medicine, University of Miami, 1500 NW 10th Avenue, Suite 807 D-56, Miami, FL 33136 USA

**Keywords:** Cushing’s disease, Recurrence, Monitoring, Pituitary, Recurrence detection

## Abstract

Transsphenoidal surgery (TSS) is first-line treatment for Cushing’s disease (CD), a devastating disorder of hypercortisolism resulting from overproduction of adrenocorticotropic hormone by a pituitary adenoma. Surgical success rates vary widely and disease may recur years after remission is achieved. Recognizing CD recurrence can be challenging; although there is general acceptance among endocrinologists that patients need lifelong follow-up, there are currently no standardized monitoring guidelines. To begin addressing this need we created a novel, systematic algorithm by integrating information from literature on relapse rates in surgically-treated CD patients and our own clinical experiences. Reported recurrence rates range from 3 to 47 % (mean time to recurrence 16–49 months), emphasizing the need for careful post-surgical patient monitoring. We recommend that patients with post-operative serum cortisol <2 µg/dL (measured 2–3 days post-surgery) be monitored semiannually for 3 years and annually thereafter. Patients with post-operative cortisol between 2 and 5 µg/dL may experience persistent or subclinical CD and should be evaluated every 2–3 months until biochemical control is achieved or additional treatment is initiated. Post-operative cortisol >5 µg/dL often signifies persistent disease and second-line treatment (e.g., immediate repeat pituitary surgery, radiotherapy, and/or medical therapy) may be considered. This follow-up algorithm aims to (a) enable early diagnosis and treatment of recurrent CD, thereby minimizing the detrimental effects of hypercortisolism, and (b) begin addressing the need for standardized guidelines for vigilant monitoring of CD patients treated by TSS, as demonstrated by the reported rates of recurrence.

## Introduction

Cushing’s disease (CD), a rare illness characterized by chronic hypercortisolemia secondary to the overproduction of adrenocorticotropic hormone (ACTH) by a pituitary adenoma, is associated with high risk of developing serious complications such as diabetes mellitus, cardiovascular disease, and depression, and a mortality rate between 1.7- and 4.8-fold higher than that of the general population [1].

The primary treatment of choice for CD is adenomectomy by transsphenoidal surgery (TSS). Surgical success rates are variable, ranging from 65 to 90 %, and depend on surgeon expertise [2, 3]. Furthermore, criteria and testing parameters for assessing immediate remission vary from center to center, making the interpretation of results challenging [4, 5]. Additionally, post-surgical recurrence of CD is not uncommon, with reported recurrence rates ranging from 3 to 47 % [6, 7], and an observed mean time to recurrence of 16–49 months [3]. For patients in whom pituitary surgery fails or is contraindicated, radiotherapy or medical treatment are alternative options.

Given the complications associated with untreated CD, it is crucial that patients receive appropriate post-surgical follow-up so that recurrence is identified and treated as early as possible. However, despite this urgency there is little published information on systematic post-surgical patient monitoring and no standardized guidelines for follow-up evaluation have been established. Moreover, patients may ultimately seek care in a wide variety of primary care and specialist settings, particularly as remission time increases, thereby necessitating a wider understanding of CD sequelae and the importance of lifelong monitoring to detect any early signs of recurrence across multiple disciplines. The aims of this review are to provide a critical overview of reported short- and long-term outcomes following surgical treatment of CD, and to propose a practical algorithm for monitoring post-surgical patients in order to detect and treat recurrence swiftly.

## Defining surgical success

The criteria for determining immediate remission following surgery are not standardized and vary widely across centers. Variability occurs in the types of biochemical parameters used to evaluate success, the assays used, limitations of the assays, and the timing of measurements [8]. Furthermore, results can be confounded by other factors, such as peri-operative administration of glucocorticoids and pre-operative treatment with steroidogenesis inhibitors [4, 5].

### Biochemical indication of remission

Early morning serum cortisol measured between 8 and 10 a.m. is the most commonly utilized measure of immediate remission following surgery. Cortisol secretion normally follows a diurnal pattern with highest levels between 7–9 a.m. that decline to lowest levels around 10–11 p.m. [9]. In patients with CD, the circadian rhythm is aberrant and cortisol levels remain consistently elevated [9, 10]. The goals of pituitary surgery are to extract the ACTH-secreting adenoma and subsequently reduce cortisol levels, leading to improvements in the signs and symptoms of CD.

Surgical success is confirmed by subnormal levels of early morning serum cortisol measured within a few days of surgery. Typically, early morning serum cortisol levels of either <2 µg/dL (~50 nmol/L) or <5 µg/dL within a few days after surgery are considered to be indicative of remission [3]. However, reported results are mixed and difficult to interpret since some studies measure basal serum cortisol levels while others use serum cortisol following dexamethasone suppression, or some combination of these measures [5, 11–13]. While clinical practice guidelines provide recommendations for testing in the context of initial diagnosis of CD [10], there is no expert consensus with regard to post-operative testing for surgical success.

The timing of biochemical measurements differs among treatment centers and can vary considerably within the same center. Intervals between surgery and biochemical measurement range from 1 to 2 days [14, 15], up to several weeks [16, 17], and even months [14]. As a result, the definition of post-surgical remission is unclear.

### Other confounding factors

This is further complicated by the fact that several circumstances can influence serum cortisol levels. Prophylactic medical treatments are often used prior to or during surgery to control cortisol levels in patients with CD. Since successful pituitary adenomectomy typically results in adrenal insufficiency, glucocorticoids are commonly administered peri- and/or pre-operatively [5]. On the other hand, because invasive surgery is a stressful procedure that elevates cortisol levels [18, 19], some centers attempt to mitigate such elevations in patients with CD, who are already hypercortisolemic as a result of the disease, prior to TSS [3, 20]. Steroidogenesis inhibitors, such as ketoconazole and metyrapone, may be administered for several weeks preceding adenomectomy [12, 21]. While the practice considers the patient’s best interest, pre-operative administration of these drugs often delays surgery and can pose challenges to the evaluation of remission status immediately following surgery. It is possible that adrenal insufficiency might be exaggerated by the action of these drugs and potentially falsely identifies some cases as surgical successes. It is therefore important for clinicians to time preoperative treatment discontinuation in order to avoid mistaking remission.

## Predicting recurrence

There are conflicting data relating to whether it is possible to identify patients who may be at greater risk for recurrence of CD following pituitary surgery. For instance, there is some evidence that tumor size, age, and gender may predict long-term outcome following surgery, and that the likelihood of recurrence may be higher in patients with macroadenomas [22–25], and patients who are younger [26]. However, a thorough review and meta-analysis of various predictive factors of recurrence found that age, gender, tumor size, and macroscopic tumor invasion were not associated with CD recurrence, whereas low levels of cortisol immediately following surgery appeared to be a positive predictor of long-term remission in more reports than not [27]. Therefore, it is unclear to what extent recurrence can be predicted, highlighting the importance of regular post-surgical patient monitoring.

Whether consideration of post-surgical adrenal insufficiency in conjunction with other parameters predicts recurrence better than post-surgical cortisol levels alone is also unclear. In one study, recovery from transient post-surgical adrenal insufficiency (2–34 months) followed by normalized hypothalamus–pituitary–adrenal (HPA) axis function predicted a low recurrence rate (13 %), whereas lack of a diurnal rhythm of cortisol secretion after normalized adrenal function predicted significantly higher recurrence rates (50–65 %) [28]. In contrast, results from another study showed that the time to complete normalization of the HPA axis following surgery was the only positive indicator of recurrence; all patients who recurred had recovered their HPA axis function within 3 years of surgery [29]. Yet others report that a lower risk of recurrence was associated with normal cortisol suppression by low-dose dexamethasone [30, 31]. Similarly, high levels of ACTH in response to corticotrophin-releasing hormone (CRH) or desmopressin stimulation better predicted relapse than post-operative serum cortisol levels alone [32–36]. As such, it is plausible that patients with subtle abnormalities of the HPA axis following surgery may be at risk for persistent or recurrent hypercortisolemia, which may be mild yet insidious.

## Subclinical CD: a conundrum

Hypercortisolemia resultant from an aberrant HPA axis can be mild, which leads to challenges with appropriately diagnosing the patient. As in most cases of subtle disease, definition of ‘subclinical’ Cushing’s syndrome (SCS), or endogenous hypercortisolemia, is somewhat ambiguous, controversial, and ill-defined. The term, almost invariably related to adrenocortical tumors, is used to describe mild hypercortisolism in the absence of the cardinal features such as violaceous striae and proximal myopathy that are commonly observed in overt Cushing’s syndrome.

Surprisingly, SCS is not a term used to call attention to the subtle hypercortisolemia commonly observed following adenomectomy for CD. Furthermore, to the best of our knowledge, there are no controlled studies focusing on the consequences of subtle or intermittent hypercortisolism in patients who have undergone surgery. This knowledge gap may, in part, be as a result of difficulties inherent to the diagnosis of mild forms of CD in the early post-operative period. Similarly, the lack of a standardized approach to evaluate the HPA axis in patients with CD following surgery may be partially due to the inconsistency and unreliability of diagnostic tests under different circumstances or severity of disease. This phenomenon, or spectrum effect, is used to emphasize this concept in which the sensitivity and specificity of tests are not fixed, but rather vary with the severity or temporal state of the disease being considered [37].

Somatic consequences of mild hypercortisolemia may be subtle or subclinical, but over time will be additive and inexorable. Evidence implies that patients may develop complications associated with long-term exposure to hypercortisolemia, such as diabetes mellitus, metabolic disturbances, and obesity [38]. More importantly, patients with SCS caused by hormonally active adrenal tumors may exhibit slow, long-term progression of these symptoms if not surgically treated. A retrospective study involving >100 patients suggested that treating SCS by means of laparoscopic adrenalectomy may improve blood pressure control, weight control, and carbohydrate metabolism [39].

In summary, persistent, subtle, autonomous ACTH secretion that engenders mild, yet continually elevated levels of cortisol is difficult to diagnose due to the presence of interfering clinical conditions, such as obesity and depression, which are more prevalent in the general population, and heterogeneity in severity or temporal stage of the disease among subgroups of examined patients. This problem can be mitigated by developing a structured and standardized approach to the evaluation of patients who undergo pituitary surgery for CD.

## Post-surgical patient monitoring

As discussed, there is no single parameter that assures permanent cure of CD. As such, a meticulous post-surgical monitoring regimen is the best approach to timely detection of recurrence. However, to date, follow-up protocols have only been reported in a handful of studies and differ from center to center [13, 15, 28]. In general, the most frequent follow-ups are done within the first year following pituitary surgery, after which monitoring frequency typically tapers off to once a year. Benefits to this follow-up protocol are that any cases of delayed remission can be identified and cases of hypocortisolism or hypopituitarism that may result from damage to the pituitary during surgery can be discovered early and treated. However, incidence of recurrence peaks within 1–5 years post-surgery with sizeable incidence also reported between 5 and 10 years of follow-up [27]. This emphasizes the need for a vigilant and uniform monitoring protocol to capture any early biochemical signs of recurrence.

## Proposed algorithm for patient follow-up

**Fig. 1:**
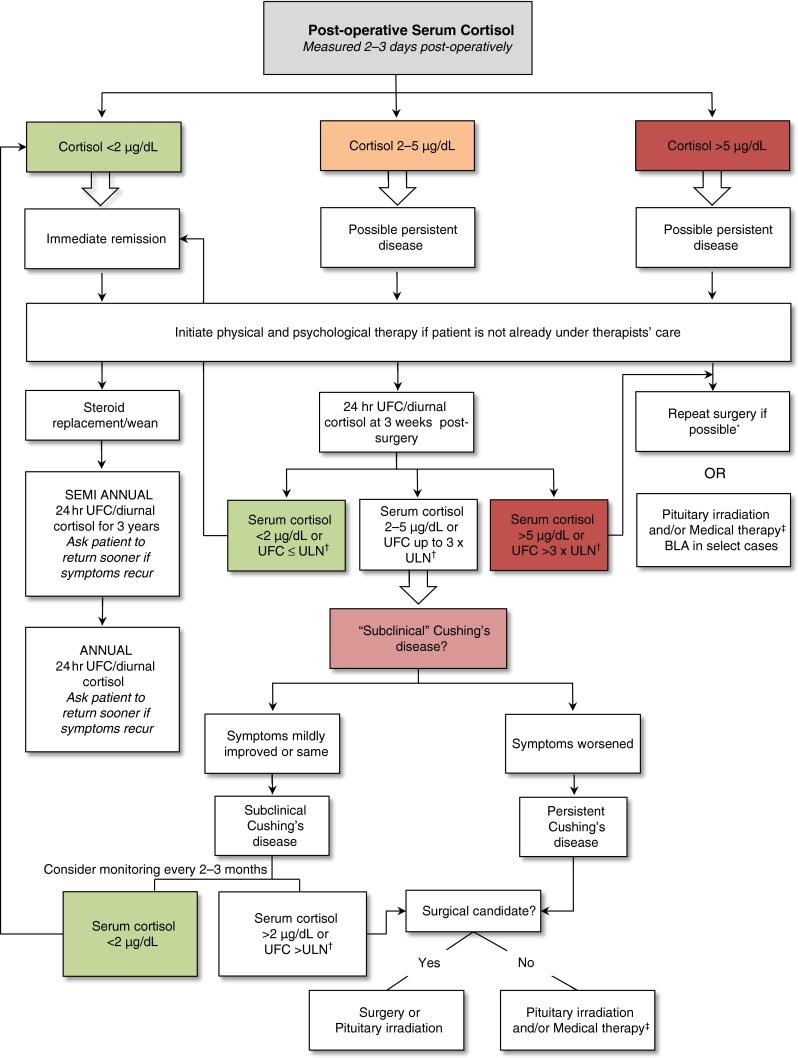
Proposed algorithm for the post-operative monitoring of patients with CD. Evaluation strategies are determined by a patient’s immediate post-surgical status as determined by early morning serum cortisol levels. *Asterisk* repeat surgery practice varies by center. Longer follow-up could be considered in patients with subnormal levels of post-operative cortisol as they may either experience delayed remission and/or may not necessarily experience recurrence. Re-intervention can be considered if cortisol levels begin to rise or if symptoms of the disease return. *Dagger* UFC upper limit of normal (ULN) as determined by specific assay used. *Double* *dagger* medical therapy: pasireotide (somatostatin analog), cabergoline (dopamine receptor agonists), mifepristone (glucocorticoid receptor antagonist), steroidogenesis inhibitors (ketoconazole, mitotane, etomidate, metyrapone)

As demonstrated, there is a lack of standardized post-surgical monitoring of patients with CD. To address this gap in clinical practice, we propose an evidence- and empirically-based algorithm to facilitate early detection and treatment of recurrence (Fig. 1). As determined by post-surgical early morning (8–10 a.m.) serum cortisol levels measured 2–3 days post-surgery, patients may be categorized as being in immediate remission (cortisol <2 µg/dL) [40, 41], potentially persistent (cortisol 2–5 µg/dL), or persistent (i.e., surgical failures; cortisol >5 µg/dL). Different courses of action are suggested for each of the three outcomes. As there is a scarcity of timing guidelines in the literature, proposed timing of evaluations is based on our own experiences.

The biochemical measure we recommend for monitoring cortisol levels is early morning serum cortisol. This is based on the following: (a) as discussed, this is the test most commonly utilized to evaluate surgical success and employing the same method ensures consistency over time, thereby providing traceable changes, and (b) it is our opinion that this is a convenient test as the patient needs only to visit the clinic to have blood drawn. Alternatively, urinary free cortisol (UFC) measurements, the most commonly used test for diagnosis of hypercortisolism, can also be used. Although assessments such as ACTH levels, CRH stimulation test, dexamethasone suppression test, and desmopressin stimulation test may be used, they are less convenient: ACTH degrades quickly and sample collection needs to be handled with great care; CRH and desmopressin stimulation tests require intravenous administration, and the dexamethasone suppression test requires the patient to take a dose of the glucocorticoid late at night and return to the clinic the next morning to have their blood drawn [42]. Although late-night salivary cortisol is useful for the screening and diagnosis of patients with CD [43], it has not been extensively studied in post-surgical patients and is therefore not currently recommended over morning serum cortisol [5].

Certain post-surgical treatment strategies apply to every patient with CD, regardless of surgical outcome. It is common for patients to experience psychiatric and physical consequences of the disease and should receive therapeutic care. The most common psychiatric manifestation of CD is depression, but anxiety, mania, and psychosis also occur [44]. Reduction of glucocorticoid action improves the system, but patients may experience significant ‘steroid withdrawal’ symptoms following remission despite adequate glucocorticoid replacement [45]. Some patients may have persistently reduced quality of life and impaired cognitive function despite long-term cure [44].

Occurrence of myopathy in patients with CD is also common and is most likely due to reduced muscle fiber conduction and decreased levels of circulating muscle proteins [46]. Osteoporosis is also a common feature of prolonged hypercortisolism [20] that leads to increased risk of bone fractures. Duration of physical and psychological therapy will depend on individual patient needs and could potentially lead to improvement in overall quality of life.

### Immediate remission cases

Immediate remission is, by definition, a condition of acute post-operative hypocortisolism. Accordingly, patients whose post-operative serum levels are <2 µg/dL will require glucocorticoid therapy. These patients should be monitored regularly to evaluate recovery of adrenal function and exogenous glucocorticoid dose should be tapered appropriately.

We propose that early morning serum cortisol levels of patients in immediate remission be monitored at semiannual intervals for 3 years. This recommendation is based on the finding that the highest rates of recurrence are observed within approximately 5 years following TSS [27]. If no elevation is observed within 3 years, monitoring frequency can be tapered to an annual basis. However, patients should be strongly encouraged to return for testing sooner if symptoms of CD begin to reappear at any point.

### Potentially persistent cases

Patients with post-operative serum cortisol levels between 2 and 5 µg/dL require much closer monitoring. These individuals are at increased risk for subclinical CD that is challenging to diagnose. We recommend that the first test be performed at 3 weeks following surgery to evaluate changes in cortisol levels (i.e., identify cases of delayed remission). If serum cortisol level declines to <2 µg/dL, the patient can be considered in remission. If serum cortisol remains elevated or if there is a mild increase in UFC values, the patient could potentially be afflicted with subclinical CD.

Careful consideration of any accompanying clinical symptoms will determine the course of action. If symptoms worsen over time, the first surgery was probably not curative and a second surgery, pituitary irradiation (radiotherapy or radiosurgery, depending on availability and the center’s practice), or medical therapy may be considered, as appropriate. If a mild elevation in serum cortisol or UFC is accompanied by either no changes or by slight improvements in clinical symptoms, the patient should be further monitored every 2–3 months. If serum cortisol levels then decrease to <2 µg/dL, the patient can be considered to be in remission. If not, treatment options, such as a second surgery, pituitary irradiation, or medical therapy may be considered if appropriate, especially if other symptoms of CD begin to reappear.

### Persistent cases

Surgery is not always curative and some patients will remain hypercortisolemic following TSS. For patients with post-operative serum cortisol >5 µg/dL, immediate repeat surgery, pituitary irradiation, or medical therapy are possible further treatment options [43, 47]. Bilateral adrenalectomy (BLA) can be an alternative in some cases, although there is a risk of the patient developing Nelson’s syndrome; BLA also necessitates lifelong glucocorticoid and mineralocorticoid replacement therapy.

## Discussion

CD is most commonly treated by surgical removal of the transgressing pituitary adenoma. Reported success rates vary widely and could be a result of surgeon expertise, reliability or timing of assays, or cut-off levels used to define remission. Furthermore, prophylactic measures, such as the use of pre- and/or peri-operative glucocorticoids or steroidogenesis inhibitors may confound results from biochemical assays performed soon after surgery [5]. It has recently been suggested that peri-operative use of glucocorticoids may be unnecessary and that they should be administered only if a biochemical or clinical need is confirmed [5]. Consideration of these variables is important because recurrence of CD following TSS is not uncommon and the current common perception is that post-surgical cortisol levels may predict a patient’s predisposition to relapse.

Despite the large number of long-term follow-up studies published, no single positive predictive factor of recurrence has emerged. Although subnormal levels of early morning serum cortisol levels measured within a few days following surgery suggest a lower risk of recurrence, relapse does occur in approximately 9 % of patients in this population [40]. Patients who achieve normal, but not subnormal post-surgical levels of cortisol are at a higher risk for recurrence (~24 %), although many of these patients may experience long-term remission [40]. Evaluation of the HPA axis in addition to cortisol levels may provide an advantage, but results to date are contradictory and further studies are needed. Since there is no fail-safe predictor of recurrence, standardized lifelong monitoring of post-surgical patients with CD is recommended for timely detection and optimal treatment of disease recurrence.

We propose the application of rigorous post-surgical monitoring to patients with CD who have been treated with TSS. Education of endocrinologists on this matter has begun with Bertagna and Guignat’s recent publication on the approach to the diagnosis and treatment of persistence and recurrent CD [48]. The authors highlight the available tests and therapeutic avenues, including combination therapies that can be used to treat a patient not cured by primary TSS. Based on our own clinical experiences and understanding of the literature, we have proposed what we believe is a feasible algorithm for post-surgical follow-up monitoring. The algorithm represents our recommendations for post-surgical monitoring of patients with CD and is presented with the caveat that it is designed to be a general guide rather than a simple protocol. Treatment decisions can be complex and must be made on a case-by-case basis using all available information, including levels of biochemical markers, severity of disease, the evolving availability of medical therapies, and patient concerns and preferences in cases of persistent hypercortisolemia.
